# Man an Object of Zoology

**Published:** 1887-09

**Authors:** S. W. Stiles

**Affiliations:** Georgia


### MAN AN OBJECT OF ZOOLOGY.

By S. W. Stiles, M. D., Ga.

[*Continued from page 310.*]

In so large a family as the monkeys we shall expect to meet with considerable varieties of form, and to find that the human character is strongly expressed in some, while others exhibit successive degrees of approximation towards the neighboring animals. They have the same number and kinds of teeth as man, and an alimentary canal very much like the human. Their pectoral mammæ and loose penis are other approximations. Linnaeus places man in the order primates of the class mammalia, and has given for companions the monkeys, the lemurs and bats. The divisions of orangs (the import of this Malay term, orang, is wild man, or man of the woods, but orang means, in fact, rational creature, and is applied to man, to the monkey in question, and to the elephant), which is the most strongly anthropo-morphous, and includes the two simiæ confounded together under the names of orang-outang, pongo, jaco, barris, and two others called gibbous, or long-armed monkeys, and S. leucisca or wouwon, is characterized by the want of a tail, by possessing an os hyoides, liver, and cæcum like the human. The S. satyrus is principally, if not altogether, found in Borneo; it is about three feet in height; the body is covered with strong reddish-brown hair. The front of the head has a very human character, the forehead being large and high, and the facial angle consequently considerable; indeed no animal approaches to man so nearly as this, in the form of the head and volume of the brain. Two large membranous bags cover the front of the neck, under the skin, and opens into the os hyoides, and thyroid cartilage, a structure which spoils him for speaking. The four component parts of the upper extremity, viz: the shoulder, arm, fore-arm and hand, can be clearly shown to exist in the anterior extremities of all mammalia, however dissimilar they may appear on a superficial inspection. The same point is illustrated in comparative anatomy, viz: the existence in an imperfect state, or anatomically speaking, as rudiments, in some animals where the function does not exist, and where the parts, therefore, are not employed. The span of the extended arms in man equals the height of the body; it is nearly double that measure in the anthropo morphous monkeys. Our upper arm is longer than the fore-arm by two or three inches; in the last mentioned animals the fore-arm is the longest. In us the hip joint divides the body equally; the lower extremity is less than half the height of the body in monkeys. In the following table I have arranged in parallel lines the dimensions of some parts of a male skeleton, and that of an orang-outang:

| | MAN. | ORANG-OUTANG. |
|---|---|---|
| | Inches. | Inches. |
| Upper ext | 32 | 25 |
| Lower ext | 39 | 16 |
| Humerus | 13 | 8½ |
| Fore-arm | 9⅞ | 9 |
| Hand | 8¼ | 7 |
| Thumb | 4½ | 3 |
| Middle-finger | 4¾ | 3 |
| Femur | 20 | 7 |
| Tibia | 16¾ | 7 |
| Foot | 10½ | 7½ |
| Middle toe | 2¼ | 2¾ |

While man is remarkable for the smoothness of his skin on the whole, some parts are even more covered with hair than in animals, as for example, the pubes and axilla, which the ancients, consequently regarded as peculiar characters of man. Other points of view, and other applications of Zoology, will be disclosed as we proceed. Enough as been said to convince enlightened readers that the living part of Nature's works is highly worthy of attention.

[To be Continued.]

